# Mrs. Garrett Anderson on Small-Pox

**Published:** 1903-09-26

**Authors:** 


The Hospital.

A Journal of

The Medical Sciences and Hospital Administration.

Vol. XXXIV.—No. 887. SEPTEMBER 26, 1903.

### Mrs. Garrett Anderson on Small-pox.

Mrs. Garrett Anderson's contribution to the history of the London small-pox epidemic of 1901–2, which appeared in the *Times* of the 15th inst. as a letter headed "Vaccination and the Rates," may well be regarded as a State paper of the first importance, deserving to be studied by all who can obtain access to it. The distinguished writer signed it in her capacity of honorary secretary to the Imperial Vaccination League; and it is therefore not unreasonable to hope that it may be issued as one of the League documents in a cheap and accessible form, and may thus be preserved from the oblivion which is apt speedily to overtake the most valuable communications to a daily paper. Mrs. Garrett Anderson takes as her text the recently issued report of the Metropolitan Asylums Board and analyses its facts and figures with especial reference to three points of supreme importance, namely, the protective influence of infant vaccination and the limits of its duration; the necessity for systematic revaccination at school age if epidemic small-pox is to be prevented; and the cost to the ratepayers of the method now employed to prevent or control epidemics of small-pox. It is proper to note that, in speaking of the enormous cost of the epidemic with which she deals, Mrs. Garrett Anderson disclaims any intention of criticising either the methods or the expenditure of the Asylums Board. She declares that the Board was given a gigantic task, and that there is no reason to think either that it failed in accomplishing it or that any other body of persons could have done better.

With reference to the first of the three questions mentioned above, the large figures dealt with by the report entirely confirm the belief that the protection afforded by infantile vaccination is at first practically complete, and that it gradually becomes exhausted during childhood and adolescence. In considering the figures of the report it is necessary to remember that vaccinated children greatly exceed the unvaccinated in number; and that both classes may be presumed to have been equally exposed to contagion. Excluding 33 children under 10, as to whom it could not be certainly determined whether they had been vaccinated or not, the report shows that in 1901 there were no deaths among vaccinated children under 10, and 65 deaths among unvaccinated. Among people under 20 who had been vaccinated 264 took the disease, of whom 175 were over 15. Of this number two died and one child between 10 and 15, making three deaths in all. Among the unvaccinated during the same period there were 95 deaths under 20, and 65 under 10. In 1902 the numbers concerned were larger, and the results still more conspicuous. There were 337 deaths among unvaccinated children under seven, and none among the vaccinated of the same age; while, as life goes on, the difference between the two classes, although always great, becomes less marked as the protective influence diminishes. In Mrs. Garrett Anderson's words, the total number of cases of small-pox varies in an imperfectly protected community with the amount of infection in each locality, but the proportion of deaths to cases varies with the degree of immunity still left to people vaccinated in infancy contrasted with the complete absence of immunity in unvaccinated people. In 1902 the percentage of deaths to cases between 15 and 20 was 2·6 in the vaccinated and 25 in the unvaccinated; between 20 and 25 it was 4·3 in the vaccinated and 31·7 in the unvaccinated; and between 25 and 30 it was 6·3 in the vaccinated and 41·2 in the unvaccinated. The great numerical majority of persons vaccinated in infancy whose protection had become so weakened as to leave them with no immunity from the disease, although with a greatly lessened liability to death, affords a sufficient explanation of the larger number of occurring cases among them than among the unvaccinated. We find that among the vaccinated under 30 there were 2,720 cases and 132 deaths, or 4·8 per cent., while among the unvaccinated there were 394 cases and 120 deaths, or 30·4 per cent. It is evident that by systematic revaccination at some stated age, which might be conveniently made to coincide with the termination of school life, so as not to interfere with earnings, the whole population might be placed in the condition of comparative safety which now exists for vaccinated children of tender years. The protective influence of vaccination does not wear out with the same rapidity after the age of childhood, with its rapid growth and active tissue changes, has passed away; and assuming the original vaccination to have been satisfactory and complete, and to have produced at least four well-formed vesicles, there is every reason to believe that a single revaccination would afford complete protection for the whole of life, except, possibly, in a few individuals of special liability who, even if they contracted the disease, would be harmless in a protected community. That this is so is sufficiently shown by the example of Germany, where revaccination is enforced by law. Germany is in direct contact with other countries in which small-pox is permitted to exist, more especially with Russia, where there were close upon thirty-three thousand deaths from the disease in 1901. In the whole of Germany in the same year there were 375 cases, with 54 deaths, 31 of these being in unvaccinated persons. The cases were distributed over 157 localities, mostly on the borders of Russia or Austria, and in no single instance did they lead to any local epidemic prevalence. We have left ourselves no space in which to deal with the financial aspects of the question, to which we shall hope to return hereafter.

